# Physiological and Full-Length Transcriptome Analyses Reveal the Dwarfing Regulation in Trifoliate Orange (*Poncirus trifoliata* L.)

**DOI:** 10.3390/plants12020271

**Published:** 2023-01-06

**Authors:** Qingqing Gu, Qingjiang Wei, Yongwei Hu, Mengru Chen, Ziwen Chen, Shuang Zheng, Qiaoli Ma, Zhengrong Luo

**Affiliations:** 1 Key Laboratory of Horticultural Plant Biology, Huazhong Agricultural University, Wuhan 430070, China; 2 College of Agronomy, Jiangxi Agricultural University, Nanchang 330045, China

**Keywords:** citrus rootstock, dwarfism, full-length transcriptome, phytohormone, carbohydrate

## Abstract

Dwarfing rootstocks are capable of high-density planting and are therefore urgently needed in the modern citrus cultivation system. However, little is known about the physiological relevance and molecular basis underlying citrus height. This study aimed to comprehensively analyze phytohormone, carbohydrate, and associated transcriptome changes in the stem of two weak growth rootstocks (‘TO’ and ‘FD’) relative to the vigorous ‘CC’ rootstock. The phenotypic observation revealed that the plant height, plant weight, and internode length were reduced in dwarfing rootstocks. Moreover, the contents of indole-3-acetic acid (IAA), trans-zeatin (tZ), and abscisic acid (ABA), were higher in TO and FD rootstocks, whereas the gibberellin 3 (GA3) content was higher in the CC rootstocks. The carbohydrate contents, including sucrose, fructose, glucose, starch, and lignin significantly decreased in both the TO and FD rootstocks. The full-length transcriptome analysis revealed a potential mechanism regulating dwarfing phenotype that was mainly related to the phytohormone signaling transduction, sugar and starch degradation, lignin synthesis, and cellulose and hemicellulose degradation processes. In addition, many transcription factors (TFs), long non-coding RNAs (lncRNAs), and alternative splicing (AS) events were identified, which might act as important contributors to control the stem elongation and development in the weak growth rootstocks. These findings might deepen the understanding of the complex mechanisms of the stem development responsible for citrus dwarfing and provide a series of candidate genes for the application in breeding new rootstocks with intensive dwarfing.

## 1. Introduction

In fruit production, dwarfism is an essential trait for high-density plantings and offers effective grove management for irrigation water and fertilizer, branch pruning, pest and disease controls, and fruit harvesting [1,2]. Grafting is commonly employed in fruit trees for asexual propagation, and rootstocks contribute to the vigor and growth of grafted trees [3,4]. Thus, using desirable rootstocks is the main way to reduce scion size and achieve dwarf cultivation. Many dwarfing rootstocks and cultivars have been used in producing apple [5,6], pear [7,8], peach [9], and some other fruit trees [10]. Nevertheless, available dwarfing germplasm resources are limited in citrus plants.

Only a few dwarfing rootstocks have been selected for citrus production. Phillips and Castle [11] evaluated the effects of 12 rootstocks on scion growth and found that ‘Rusk’ citrange and various trifoliate oranges, particularly ‘English small’, induced the semi-dwarf tree size of orange plants. Three hybrid rootstocks ‘FA517′, ‘FA418′, and ‘#23′ were reported as new dwarfing citrus rootstocks, which could inhibit scion growth by reducing water and carbohydrate transport, and enhancing reproductive development [12,13,14]. Liu et al. [15] revealed that the ‘Shatangju’ mandarin grafted onto Fragrant orange and Red tangerine rootstocks showed dwarfing characteristics, as indicated by the short length of shoots and internodes, small trunk diameter of the scions, and reduced growth vigor. Trifoliate orange (*Poncirus trifoliata*, TO) is the most frequently used citrus rootstock in China and has become naturalized in other regions of Asia, Australia, Europe, and the USA [16,17]. In China, TO species have been divided into polyploids, thornless type, large-leaf (flower) type, and small-leaf (flower) type [18]. Citrus plants grafted onto the small-leaf (flower) type exhibit relatively small tree sizes [19]. Additionally, TO rootstock possesses various merits, including good grafting compatibility and favorable adaptation to different abiotic/biotic constraints [20,21,22]. Despite the advantageous characteristics of trifoliate orange, new dwarfing rootstocks are highly desirable for new scion varieties and modern citrus production. ‘Flying dragon’ trifoliate orange (*P. trifoliata* var. *monstrosa*, FD), a mutant derived from trifoliate orange, is considered to have similar horticultural traits but intensive dwarfing compared with the TO standard rootstock [23,24,25]. The height of adult trees grafted on FD is not more than 2.5 m [26]. A recent study showed that the ‘Shatangju’ mandarin scion grafted onto FD rootstock tends to be dwarfing and develops short-stature plants [27]. However, the molecular basis associated with plant height in FD remains largely unexplored, although this information is potentially useful for evaluating FD as a breeding parent for new rootstocks.

Dwarfing in plants is a complicated developmental process, involving a series of anatomical, physiological, biochemical, and hormonal changes. Studies have shown that many phenotypic adjustments, such as shortened internode length, decreased internode numbers, abnormal cell walls, or cell elongation, facilitate plant dwarfism [28,29]. For instance, the anatomical analysis of peach and pear rootstocks revealed that the dwarfing types had smaller xylem vessel diameters and densities than the standard types [30,31]. Zorić et al. [32] demonstrated that the dwarfing rootstocks had a smaller vessel lumen size and lower theoretical hydraulic conductance than vigorous rootstocks in cherry. Besides phenotypes, alterations in various metabolisms are closely related to plant dwarfism. In apples, dwarfing rootstocks (‘M27′ and ‘M9′) had a higher starch content but lower glucose and fructose contents than the vigorous rootstock ‘M793′ [33]. The metabolome analysis showed that a series of metabolites, including flavonoids, lignans, phenolic acids, and amino acids and their derivatives were highly accumulated in the pseudostem of a semi-dwarf banana mutant [34]. Cui et al. [35] suggested that dwarfing ‘OHF51′ as an interstock changed the metabolite concentrations of the scion and rootstock, and the flavonoids and phenolic acids/derivatives were key compounds implicated in the control of scion growth. Recently, more attention has been paid to characterizing the role of phytohormones in regulating plant dwarfing. The reduction in the levels of growth-promoting hormones such as auxins, cytokinins (CKs), brassinosteroids (BRs), and gibberellins (GAs), and the increase in the levels of growth-inhibiting hormones such as jasmonic acid (JA), salicylic acid (SA), and abscisic acid (ABA) are important causes of plant dwarfing [10]. Many dwarfing-related genes from the phytohormone biosynthesis and signal transduction, cellulose and fiber elongation, and transcriptional regulation pathways have been identified and functionally analyzed [36,37,38], providing a theoretical basis for the use of candidate genes to enhance plant dwarfing.

The availability of the trifoliate orange genome [21], has made it possible to gain a comprehensive insight into the cellular and genetic mechanisms regulating dwarfing phenotype. In addition, third-generation sequencing technologies, such as Oxford Nanopore Technology (ONT), provide huge advantages in lncRNA prediction, transcript identification, and transcript expression quantification in plants [39]. Thus, an excellent opportunity exists for identifying the genetic contributors to different agronomic traits in plants using ONT sequencing. The present study aimed to explore biological processes and gene regulatory networks in the stem of the TO belonging to the small-leaf (flower) type and dwarfing FD rootstocks relative to the vigorous rootstock ‘Carrizo’ citrange (*Citrus sinensis* × *P trifoliata*, CC). We compared the phytohormone and carbohydrate changes between the weak and vigorous rootstocks. We also generated nine full-length transcriptomes of CC, TO, and FD rootstocks using ONT sequencing and identified differentially expressed genes (DEGs) and regulatory factors in the stem of these three rootstocks. The results of the present study might increase the understanding of the molecular mechanism involved in the dwarfing trait of citrus plants.

## 2. Results

### 2.1. Comparison of Phenotypic Characteristics among Three Rootstocks

**Figure 1:**
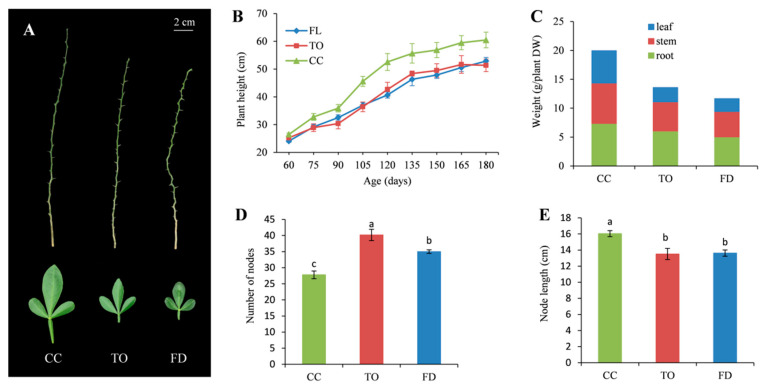
Stem and leaf morphology (**A**), dynamic change in plant height (**B**), weight of different parts of the plant (**C**), number of nodes in the stem (**D**), and node length (**E**) of the CC, TO, and FD rootstocks. Different small letters indicate significant differences at *p* < 0.05 according to Duncan’s multiple range tests.

The CC plants always exhibited more vigorous growth than the TO and FD plants (Figure 1A). The TO and FD rootstocks showed differences in plant height compared with the CC rootstocks during 60–180 days of growth (Figure 1B). After 180 days of growth, the three rootstocks presented significant changes in other growth parameters. The weights of the leaf, stem, and root parts were significantly lower in the TO and FD plants (Figure 1C). Regarding the number of nodes in the stem, the values associated with TO were the largest, followed by FD and CC (Figure 1D). However, the CC rootstocks supported longer internode lengths than the TO and FD rootstocks (Figure 1E).

### 2.2. Summary of Full-Length RNA-Seq and Characterization of Transcripts and Alternative Splicing Events

Full-length single-molecule transcriptomes of the young stem were generated from three citrus rootstocks to explore the molecular mechanisms determining growth performance. From the initial 36,776,306 raw reads, 36,665,318 (99.70%) clean reads were generated, with N50 ranging from 1117 to 1263 and mean lengths ranging from 1050 to 1113 bp (Table S1). Among these clean reads, 31,427,894 full-length reads with clear primer sequences at both ends were identified. The full-length reads comprised 84.18% (3,963,437; FD2) to 87.14% (3,742,087; TO1) of the clean reads. All full-length reads were clustered and polished to yield consensus reads. All the consensus reads were then aligned to the *Poncirus trifoliata* genome with mapping rates of more than 98.56% to remove redundant reads (Table S1).

**Figure 2:**
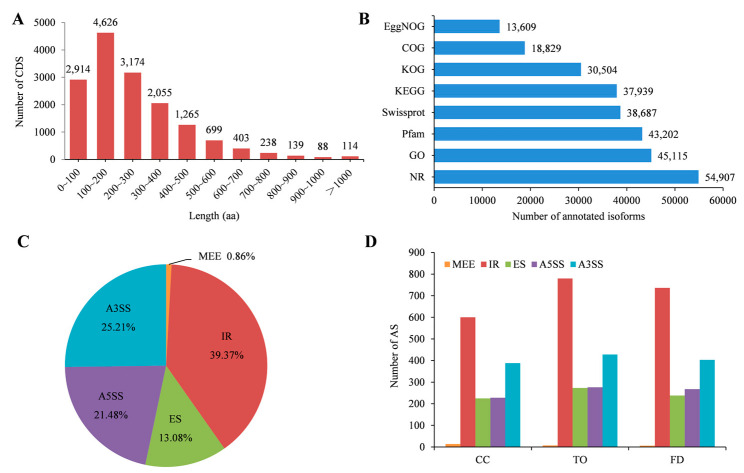
Characteristics of the transcripts and alternative splicing events. (**A**): Length distribution of all predicted peptides. (**B**): Functional annotations of new isoforms using eight different databases. (**C**): Summary of all alternative splicing events. (**D**): Alternative splicing events in the CC, TO, and FD rootstocks.

Moreover, 15,715 open reading frames were identified. Of these, 62.72% of the coding sequences (CDSs) encoded for small peptides comprising 100–400 amino acids (aa), and only 114 CDSs were encoded for >1000 aa (Figure 2A). Finally, 55,021 transcript sequences were obtained by searching against eight different databases. As shown in Figure 2B, 13,609, 18,829, 30,504, 37,939, 38,687, 43,202, 45,115, and 54,907 new isoforms were annotated to the EggNOG, COG (Clusters of Orthologous Groups), KOG (euKaryotic Ortholog Groups), KEGG (Kyoto Encyclopedia of Genes and Genomes), Swissprot, Pfam, GO (gene ontology), and NR (NCBI non-redundant protein sequence) databases, respectively. 

Additionally, 5260 alternative splicing (AS) events were identified from the transcripts in the nine libraries of the CC, TO, and FD rootstocks (Table S2). Among the five main types of AS, 39.31% were intron retention (IR), 25.21% were alternative 3’ splice site (A3SS), 21.48% were alternative 5′ splice site (A5SS), 13.08% were exon skipping (ES), and only 0.86% were mutually exclusive exon (MEE) (Figure 2C). Moreover, the distribution of these five types of AS events in each rootstock was different. A total of 780 and 736 IR events occurred in the TO and FD rootstocks, and 600 in the CC rootstocks (Figure 2C).

### 2.3. Comparative Analysis of DEGs

**Figure 3:**
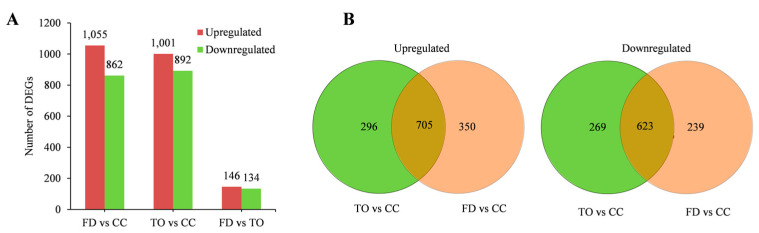
DEGs in the stem of three rootstocks. (**A**): The numbers of upregulated and downregulated DEGs in the indicated comparisons. (**B**): Venn diagrams show the overlapped DEGs in the TO and FD rootstocks compared with the CC rootstocks.

A total of 1893 DEGs between TO and CC, and 1917 DEGs between FD and CC were identified using an adjusted *p*-value of 0.05 and |log2 fold change|> 1 as a cutoff, with 1001 upregulated and 892 downregulated genes, and 1055 upregulated and 862 downregulated genes, respectively. In contrast, only 280 DEGs were detected between TO and FD rootstocks (Figure 3A). The relationships among different DEG groups were displayed as Venn diagrams. Further, 1328 DEGs overlapped in FD and TO rootstocks compared with the CC rootstocks, with 705 being upregulated and 623 downregulated (Figure 3B). 

GO annotation and KEGG pathway classification were performed to further explore the function of these DEGs common to both TO and FD rootstocks. These DEGs were enriched in 45 GO terms, which could be divided into biological processes, cellular components, and molecular functions. Of these, the top five subcategories were catalytic activity (454), binding (417), membrane (314), metabolic process (309), and cellular process (302) (Figure S1). The KEGG analysis revealed that 710 genes were assigned to 118 pathways. A majority of them were metabolism-related pathways, including phenylpropanoid biosynthesis (35), starch and sucrose metabolism (20), and carbon metabolism (18). Additionally, 36 and 35 DEGs were involved in plant hormone signal transduction and plant-pathogen interaction pathways, respectively (Figure S2). 

### 2.4. Analysis of Phytohormone Contents and the Related Gene Expression

**Figure 4:**
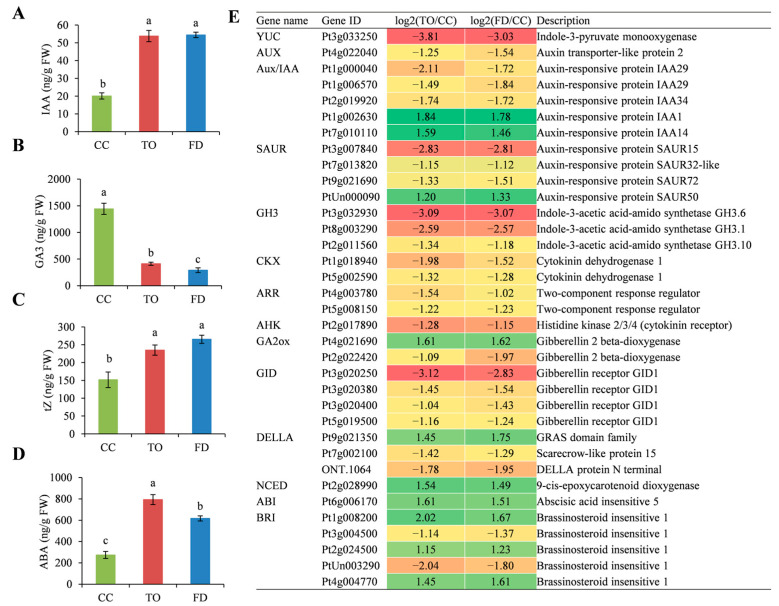
Changes in the content of IAA (**A**), tZ (**B**), GA3 (**C**), and ABA (**D**), and their metabolism-related gene expression (**E**). Different small letters indicate significant differences at *p* < 0.05 according to Duncan’s multiple range tests.

Given that hormones play key roles in plant growth regulation, the indole-3-acetic acid (IAA), trans-zeatin (tZ), gibberellin 3 (GA3), and ABA contents in the young stems of the three rootstocks were compared (Figure 4A–D). The result showed that IAA and tZ concentrations were similar between TO and FD, and significantly higher than those in the CC rootstocks. In contrast, GA3 contents in the CC rootstocks were approximately 3.5-fold and 5-fold higher than those in TO and FD rootstocks, respectively. On the whole, the concentrations of IAA, tZ, and GA, which are often thought to be growth-promoting factors in plants, were significantly higher in the weak TO and FD rootstocks than in the vigorous CC rootstocks. Nevertheless, the ABA content was the highest in the TO rootstocks, followed by the FD, and CC rootstocks.

Subsequently, a detailed analysis of DEGs was conducted under the phytohormone synthesis and transduction pathway. A total of 35 DEGs were identified and most of them were downregulated in both the TO and FD rootstocks compared with the CC rootstocks (Figure 4E). Further, 14 genes were associated with auxin biosynthesis and transduction. Of these, one *indole-3-pyruvate monooxygenase* (*YUC*) gene and one *AUX* gene (Pt4g022040) showed significantly lower expression levels in the TO and FD rootstocks than in the CC rootstocks. Besides, the expression of early auxin response genes also changed greatly among these rootstocks. Three of five *auxin/indole-3-acetic acid* (*Aux/IAA*) genes, three of four *Small auxin up-RNA* (*SAUR*) genes, and all three *Gretchen Hagen3* (*GH3*) genes showed downregulation in the dwarf rootstocks. Five cytokinin-related genes, including two *cytokinin oxidase/dehydrogenase* (*CKX*) genes, two *Arabidopsis response regulators* (*ARR*) genes, and one *Arabidopsis histidine kinase* (*AHK*) gene were identified, and all these genes were expressed at lower levels in the TO and FD rootstocks than in the CC rootstocks. Regarding gibberellins, two *gibberellin 2 beta-dioxygenase* (*GA2ox*) genes were identified, of which one (Pt4g021690) was upregulated and the other (Pt2g022420) was downregulated. All four *gibberellin insensitive dwarf* (*GID*) members were downregulated. In addition, two out of three genes encoding the DELLA protein were downregulated. In contrast, both *9-cis-epoxycarotenoid dioxygenase* (*NCED*) and *abscisic acid insensitive* (*ABI*) genes involved in ABA biosynthesis were significantly upregulated in the TO and FD rootstocks. Furthermore, seven genes related to other phytohormone metabolism and signaling processes were also screened. Five *brassinosteroid insensitive* (*BRI*) genes demonstrated significantly differential expression, of which two (Pt3g004500, PtUn003290) were downregulated and the other three (Pt1g008200, Pt2g024500, Pt4g004770) were upregulated (Figure 4E).

### 2.5. Analysis of Sugar Contents and the Related Gene Expression

**Figure 5:**
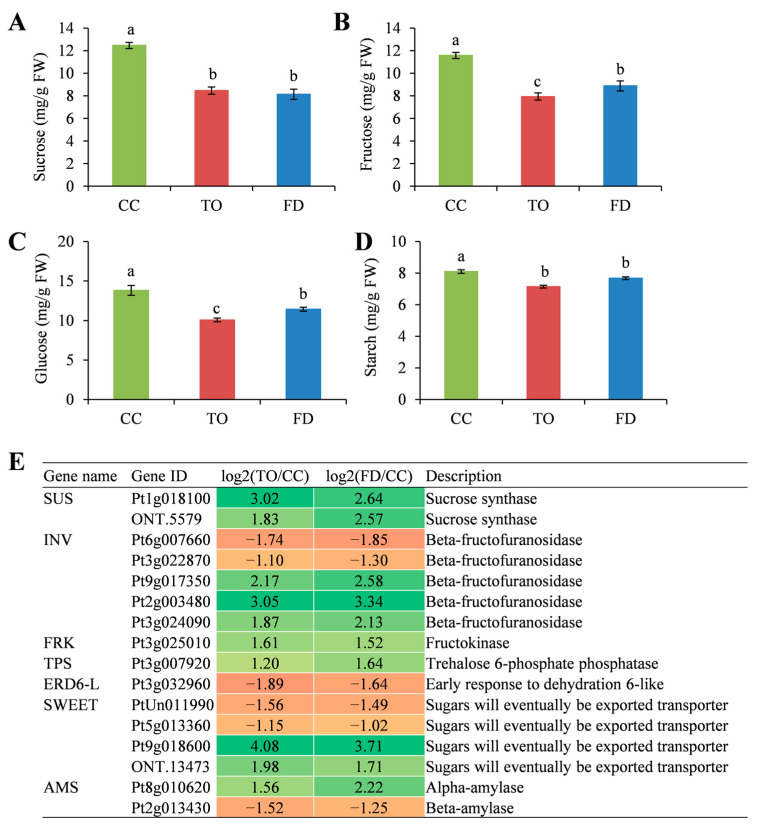
Changes in the content of sucrose (**A**), fructose (**B**), glucose (**C**), starch (**D**), and their metabolism-related gene expression (**E**). Different small letters indicate significant differences at *p* < 0.05 according to Duncan’s multiple range tests.

Carbohydrate metabolism is considered to be closely related to plant cell formation and development. The determination of nonstructural carbohydrates, including sucrose, fructose, glucose, and starch, showed that these sugar metabolites changed among the three rootstocks. The sucrose concentration was similar in the TO and FD rootstocks, but both showed lower levels than the CC rootstocks (Figure 5A). Like sucrose, the starch content showed no significant difference in the TO and FD rootstocks, but it was higher in the CC rootstocks (Figure 5D). The present study also found that glucose was the dominant component, and all analyzed carbohydrates had the highest concentration in the CC plants, followed by the FD and TO plants (Figure 5B,C).

The expression levels of genes implicated in the starch and sucrose metabolism pathway were assessed, and it was found that many sugar-metabolism-related genes, including *sucrose synthase* (*SUS*), *beta-fructofuranosidase* (*INV*), *fructokinase* (*FRK*), *trehalose 6-phosphate phosphatase* (*TPS*), *early response to dehydration 6-like* (*ERD6-L*), and *sugars will eventually be exported transporter* (*SWEET*) were differentially expressed among the three rootstocks (Figure 5E). *SUS* (Pt1g018100, ONT.5579), *FRK* (Pt3g025010), and *TPS* (Pt3g007920) genes were upregulated in the TO and FD plants compared with the CC plants. Likewise, three out of five *INV* genes showed upregulation in TO and FD rootstocks. The genes related to sugar transport were also screened out. One *ERD6-L* (Pt3g032960) showed downregulation. The expression of *SWEET* genes changed greatly, and three out of five members were upregulated in the TO and FD rootstocks. Specifically, the expression level of one *SWEET* (Pt9g018600) was upregulated by 4.08-fold in the TO and 3.71-fold in the FD compared with the CC rootstocks (Figure 5E). Regarding starch metabolism, only two *amylase* (*AMS*) genes were found, with one *alpha-amylase* member (Pt8g010620) upregulated and one *beta-amylase* member (Pt2g013430) downregulated.

### 2.6. Analysis of Cell Wall Components and the Related Gene Expression

**Figure 6:**
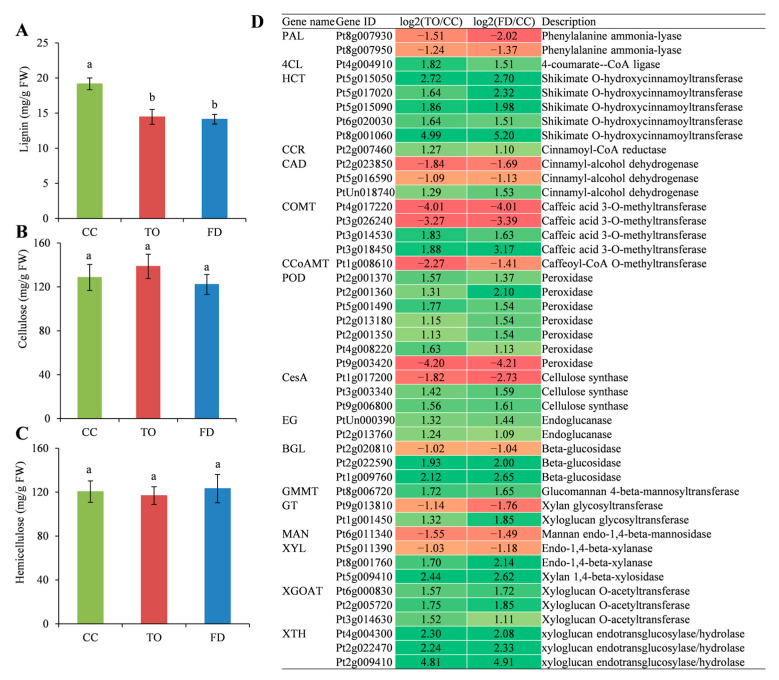
Changes in the content of lignin (**A**), cellulose (**B**), hemicellulose (**C**), and their metabolism-related gene expression (**D**). Different small letters indicate significant differences at *p* < 0.05 according to Duncan’s multiple range tests.

Lignin, cellulose, and hemicellulose are the dominant components of cell walls. Our results showed that the lignin content was significantly reduced in the TO and FD rootstocks (Figure 6A). However, no significant difference in both cellulose and hemicellulose contents was observed among the three rootstocks (Figure 6B,C).

Furthermore, the study analyzed the DEGs belonging to lignin, cellulose, and hemicellulose metabolism pathways. The result showed that 24 DEGs related to the phenylpropanoid pathway were regulated (Figure 6D). Of these, two *phenylalanine ammonia-lyase* (*PAL*) genes were downregulated in the TO and FD plants. However, all five *shikimate O-hydroxycinnamoyltransferase* (*HTC*) genes and one *4-coumarate-CoA ligase* (*4CL*) were upregulated. Additionally, six out of seven *peroxidases* (*POD*) genes were upregulated, except for one member (Pt9g003420), which was largely downregulated by 4.2-fold in the TO and FD seedlings. One *cinnamoyl-CoA reductase* (*CCR*) gene showed upregulation, whereas one *caffeoyl-CoA O-methyltransferase* (*CCoAMT*) gene showed downregulation in both TO and FD rootstocks. Moreover, the expression of *cinnamyl-alcohol dehydrogenase* (*CAD*) and *caffeic acid 3-O-methyltransferase* (*COMT*) genes differentially changed, with two of three and two of four downregulated members, respectively.

Besides, the genes related to cellulose and hemicellulose metabolisms, such as *cellulose synthase* (*CesA*), *endoglucanase* (*EG*), *Beta-glucosidase* (*BGL*), *glucomannan 4-beta-mannosyltransferase* (*GMMT*), *glycosyltransferase* (*GT*), *mannan endo-1,4-beta-mannosidasee* (*MAN*), *endo-1,4-beta-xylanase, xylan 1,4-beta-xylosidase* (*XYL*), *xyloglucan O-acetyltransferase* (*XGOAT*), and *xyloglucan: xyloglucosyl transferase* (*XTH*), were identified. It was observed that two of three *CesA* genes demonstrated upregulation in the TO and FD rootstocks. Both *EG* and *BGL* genes implicated in cellulose degradation were also upregulated, except for one *BGL* member (Pt2g020810), which was slightly downregulated in the TO and FD (Figure 6D). For hemicellulose, three synthesis-related genes were identified, of which *GMMT* (Pt8g006720) and *GT* (Pt1g001450) genes were upregulated, and the *GT* (Pt9g013810) was downregulated. Meanwhile, 10 DEGs related to hemicellulose degradation were also identified. Except for one *MAN* (Pt6g011340) and *XYL* (Pt5g011390), the expression of the other eight genes, including three *XGOAT* genes, three *XTH* genes, and two *XYL* genes, showed higher levels in the TO and FD rootstocks than in the CC rootstocks (Figure 6D).

### 2.7. Characterization of the Transcription Factors and Long Noncoding RNAs 

**Figure 7:**
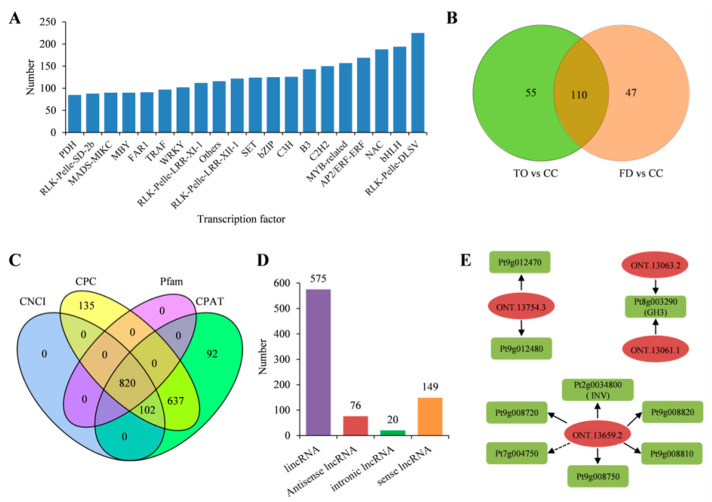
Identification of TFs and lncRNAs based on the full-length transcriptome. (**A**): Numbers of the top 20 TF families identified in all full-length transcripts. (**B**): Venn diagram shows the overlapped in the differential expressed FTs among the three rootstocks. (**C**): Venn diagrams show the number of lncRNAs identified using four different public databases. (**D**): Number of four lncRNA types. (**E**): Examples of predicted interaction networks among lncRNAs and their target genes. Solid lines and dotted lines indicate the expression regulation by the lncRNAs in cis and trans, respectively.

Transcription factors (TFs) are involved in transcriptional regulatory networks and are known to be associated with plant growth and development. In the present study, a total of 5979 TFs were detected (Table S3). The top 20 TF families identified are shown in Figure 7A. Of these, the *RLK-Pelle-DLSV*, *bHLH*, and *NAC* families constituted the most abundant TFs. Furthermore, 165 and 157 TFs were differentially expressed in the TO and FD rootstocks, respectively, compared with the CC plants. Among these, 110 differential TFs overlapped between these two rootstocks, with 58 upregulated and 52 downregulated members (Figure 7B). We further investigated these common TFs and found that all *B3* and *bHLH* genes and most *C2C2* and *MYB* genes were upregulated, whereas most *GNAT*, *GRAS*, *NAC*, *TRAF*, *bZIP*, *AP2/ERF-ERF*, *RLK-Pelle-LRR-X*, and *RLK-Pelle-DLSV* genes were downregulated in the TO and FD rootstocks. Of these genes, the expression levels of *CMGC-CDK-Pl* (PtUn016980) and *RLK-Pelle-LRR-I-1* (Pt7g011350) genes displayed the lowest and highest fold changes in the TO and FD rootstocks than those in CC rootstocks, respectively (Table S3).

Long noncoding RNAs (lncRNAs) are non-coding transcripts that regulate both cis- and transgene transcription in plants. Here, four public databases including CPC, CNCI, CPAT, and Pfam were combined to sort lncRNAs, and 820 annotated transcripts were found in all the datasets (Figure 7C). Among these common lncRNAs, lincRNAs predominated, accounting for 70.1%, followed by sense lncRNAs (18.2%), antisense lncRNAs (9.3%), and intronic lncRNAs (2.4%) (Figure 7D). Furthermore, 16,014 target genes for 819 lncRNAs were predicted. Of these target genes, 830 and 15,184 were regulated by lncRNAs in trans and cis, respectively (Table S4). The networks of several target genes and their corresponding lncRNAs are shown in Figure 7E. In detail, Pt2g0034800 (*INV*) and the other five genes were the target genes of lncRNA ONT.13659.2, Pt9g012470, and Pt9g012480 were the target genes of lncRNA ONT.13754.3, and Pt8g003290 (*GH3*) was the target gene of lncRNAs ONT.13061.1 and ONT.13063.2.

## 3. Discussion

Dwarfing rootstocks plays a key role in simplifying orchard management and reducing production costs. Therefore, producing superior dwarfing rootstocks for fruit trees and exploring the basic mechanism of plant dwarfing are of great importance. Previous studies found that dwarfism was often correlated with changes in stem elongation and related cellular processes [29,40]. In this study, the node number increased but the node length, plant height, and biomass decreased in the TO and FD rootstocks. Although FD was considered to be more dwarfing, it showed no difference in plant height and node length compared with the TO rootstocks. Overall, our results suggested that both dwarfing rootstocks, specifically the FD rootstocks, exhibited a distinct pattern in stem elongation, and they could be appropriate materials for further mechanistic elucidation and germplasm construction for citrus dwarfing.

### 3.1. Phytohormones Influence Stem Elongation

To date, most dwarfing traits discovered in plants have been associated with changes in phytohormone metabolism. IAA, tZ, and GA can promote cell division and elongation in plant tissues, and act as growth-promoting factors [10]. In contrast, the ABA concentration in the tissues of dwarfing apple and citrus trees is higher than that in vigorous varieties, and it could inhibit plant growth by suppressing the accumulation of other endogenous hormones [22,41]. The TO and FD rootstocks accumulated more IAA, tZ, and ABA. Meanwhile, GA was the dominant phytohormone and showed a lower level in the stems of the TO and FD rootstocks compared with the CC rootstocks. Finally, the TO and FD rootstocks had a lower (IAA + tZ + GA3)/ABA ratio than the CC rootstocks. Similar results were obtained in apple and citrus plants [6,19]. It was suggested that a balance among hormones but not only a single hormone exerts a great influence on overall citrus rootstock growth. This study revealed that phytohormone signal transduction was significantly enriched, and the DEGs were mainly related to auxin and GA signaling pathways. Auxin is synthesized from tryptophan in plants depending on a series of enzymes such as TAA and YUC and then transported between plant cells in coordination with two kinds of carrier proteins, namely the auxin influx carriers belonging to the AUX1/LAX protein family and the auxin efflux carriers belonging to the PIN protein family [42]. In TO and FD rootstocks, we found that both *YUC* and *AUX* genes were downregulated. In addition, the *Aux/IAA*, *SAUR*, and *GH3* genes, which are the three major classes of auxin response genes, were also mostly downregulated in the TO and FD rootstocks. The *Aux/IAA* gene family includes important transcriptional inhibitors during the auxin signaling processes, which can respond to auxin and rapidly degrade, thereby initiating the auxin signal transduction pathway [43]. GH3 proteins catalyze endogenous IAA to conjugate with amino acids and deactivate IAA to reduce the auxin signal [44]. SAUR proteins can serve as inhibiting or promoting factors in plant growth and development. A total of 79 *SAUR* genes have been found in Arabidopsis thaliana, of which *SAUR19* and *SAUR63* are positive regulators of cell growth. When the expression level of *SAUR19* was reduced by microRNA interference, the seedlings showed shorter hypocotyls and smaller leaf areas [45]. Therefore, the downregulation of *Aux/IAA*, *SAUR*, and *GH3* genes may perturb the auxin signaling transduction in the TO and FD rootstocks, and partly contribute to their weak growth. Gibberellins constitute a large group of diterpenoid carboxylic acids. Some of them are bioactive and regulate multiple aspects of plant growth and development. GA is synthesized by the action of terpene cyclases, cytochrome P450 mono-oxygenases, and 2-oxoglutarate-dependent dioxygenases, and its deactivation is catalyzed by GA2ox in higher plants [46]. The overexpression of *GA2ox* decreased the content of bioactive GAs in transgenic plants, producing dwarf phenotypes [47,48]. In this study, no DEGs related to GA synthesis were identified among these rootstocks. However, two *GA2ox* genes showed differential expression, indicating their important roles in growth regulation in citrus rootstocks via disturbing GA metabolism. The GA signaling pathway contains three key components, namely GID1, GID2, and DELLA. Gibberellins bind within a pocket of GID1, causing a conformational change that facilitates the formation of a GA-GID1-DELLA complex and the subsequent ubiquitination of DELLAs and their destruction by the 26S proteasome [46]. *GID1* and *DELLA* genes generally had lower expression in the TO and FD citrus rootstocks compared with the CC rootstocks. Hence, it was indicated that the disordered change in the expression levels of genes related to GA signaling transduction was adverse for citrus growth, which was an important reason for the dwarfing traits of TO and FD plants.

Additionally, RNA-seq showed that various DEGs, including *CKX* genes associated with cytokinin degradation, and *ARR* and *AHK* genes associated with cytokinin signal transduction [49,50], were significantly downregulated, suggesting that cytokinins were also involved in regulating stem elongation in citrus. However, the genes implicated in ABA and BR pathways were found to be mostly upregulated in this study. These genes may influence the corresponding phytohormone metabolism and signaling pathways and are possibly implicated in complex networks of phytohormone processes that participate in the coordination of cell division and cell elongation, resulting in the overall shortened stem and decreased plant height in the TO and FD rootstocks.

### 3.2. Involvement of Carbohydrate Metabolism in Dwarfing Regulation

Carbohydrates have central regulatory roles in maintaining normal physiological processes in plants. Varying with the species and cultivar, stem carbohydrates are mainly stored as soluble sugars, such as sucrose, starch, or fructans [51]. Accumulating evidence has suggested sugars act as not only nutrients but also signaling molecules that contribute to sensing the nutrient status and coordinating growth and development in plants [52]. For instance, sugars promote cell cycle progression in starved cell cultures and seedlings by inducing the expression of cyclins *CycD2* and *CycD3* in Arabidopsis [53]. In the present study, the accumulation of sucrose, fructose, glucose, and starch in the stem was lower in the TO and FD rootstocks than that in vigorous CC rootstocks, which would have significant impacts on the stem growth of these rootstocks. Consistently, a set of key genes participating in starch and sucrose metabolism were screened, among which the expression of *SUS*, *INV*, *FRK*, *TPS*, and *AMS* genes was mostly induced in the TO and FD rootstocks, indicating that the sugar and starch degradation might be activated in the dwarfing rootstocks. Our results were in line with previous findings that the dwarfing apple rootstocks presented an imbalance of carbohydrate allocation as indicated by the decrease in glucose, fructose, and *Myo*-inositol contents and the downregulation of the related genes [33]. Plant sugar transporters, mainly SWEET, ERD6-L, SUT, and TST facilitate the allocation of various sugars at both the organismal and the cellular levels [54]. As indicated in the present study, we screened out one *ERD6-L* and four *SWEET* genes that might control sugar storage and accumulation in citrus rootstocks. Among these, one *SWEET* member (Pt9g018600) increased its transcript abundance and was potentially involved in sugar transport in the FD and TO plants responsible for their growth.

Other carbohydrates such as lignin, cellulose, and hemicellulose are the dominant components of cell walls. The synthesis of these components is crucial for the mechanical properties of growing cells. In Arabidopsis, the mutants defective in lignin synthesis pathway genes often exhibited reduced lignin content and severe dwarf phenotype, which was referred to as lignin-modification-induced dwarfism (LMID) [55]. Other reports showed that plant dwarf phenotype was often related to the regulation of various genes in the lignin biosynthetic pathway. For example, Wang et al. [29] found the *PAL*, *4CL*, *C3H*, *F5H*, *HCT*, and *CCR* genes promoting the biosynthesis of the three monolignols showed downregulation in the basal internodes of the dwarfing bamboo, whereas *POD* and *laccase* genes responsible for the oxidation and polymerization of monolignols were upregulated in the basal and middle internodes. We also found that eight lignin biosynthetic genes, particularly the *COMT* (Pt4g017220, Pt3g026240) and *POD* (Pt9g003420), showed significantly reduced expression levels in the TO and FD compared with the CC rootstocks. The downregulation of these genes in dwarfing citrus rootstocks indicates a reduced lignin accumulation. This was further supported by the observation that the lignin content was reduced in the TO and FD rootstocks (Figure 6A). Cellulose consists of β-1,4-linked glucan residue chains between 500 and 14,000 monomers long, whereas hemicellulose has β-1,4-linked backbones that include xylans, xyloglucans, mannans, and their derivatives. Hemicelluloses tether cellulose microfibrils in the primary cell wall, strengthening the cell wall and limiting cell enlargement [56,57]. In this study, both cellulose and hemicellulose contents showed no difference among the stems of CC, TO, and FD rootstocks. However, various genes involved in cellulose/hemicellulose metabolisms showed differential expression between the weak and vigorous rootstocks (Figure 6D). The *CesA, cellulose synthase-like* (*Csl*), *GMMT*, and *GT* were reported to be responsible for the biosynthesis of cellulose, hemicellulose, and pectin polysaccharides of the plant cell wall, and they provide mechanical strength for plant cells and coordinated signaling between the newly elongated tissues [56,58].

Thus, the downregulated expression profiles of *CesA* (Pt1g017200) and *GT* (Pt9g013810) genes in the TO and FD rootstocks might limit cell wall biosynthesis. Meanwhile, the results revealed that multiple genes involved in cellulose and hemicellulose degradation, including *EG* and *BGL* genes belonging to the complex cellulase gene superfamily [59], *XYL* genes mediating xylan hydrolysis, and *XGOAT* and *XTH* genes mediatingxyloglucan hydrolysis [57,60], were mostly upregulated in both the TO and FD rootstocks compared with the CC rootstocks. This might facilitate cell wall hydrolysis and catabolism and, finally, inhibit tissue fiber elongation in the TO and FD rootstocks.

### 3.3. Identification of Regulatory Factors Associated with Plant Growth

Before the regulation of functional gene transcript diversity during plant growth, specific TFs play a key role at the transcriptional level. Various TFs such as WRKY, NAC, AP2/EREBP, C2H2, MYB, and bHLH are known to be important for plant development and dwarfing [37,61,62]. Based on the ONT-sequencing data, 110 differentially expressed TFs were identified. Among these, bHLH was highly represented in the transcriptome results, and all eight identified members were upregulated in the TO and FD rootstocks. Previous studies showed that bHLH genes were associated with tissue development and abiotic stress [63,64]. Thus, it was concluded that the bHLH TFs played positive regulatory roles in the dwarf phenotype of citrus plants. Interestingly, many other TFs such as *CMGC-CDK-Pl* annotated as cell division protein kinase and *RLK-Pelle-LRR-I-1* annotated as leucine-rich repeat receptor-like protein kinase also exhibited significant changes in their expression levels, indicating that they were also key regulatory factors that activated or suppressed the transcription of multiple genes during primary and secondary growth. However, whether these TFs can cause dwarfism in plants requires further investigation.

LncRNAs, in sync with FTs, are important components of gene regulatory networks that participate in plant tissue development. Many lncRNAs that are implicated in stem development have been identified in *Populus* [65]. Another study showed that lncRNAs might be tissue- and developmental stage-specific and associated with leaf, inflorescence, and berry tissue development in grapevine [66]. Our results also revealed that lncRNAs targeted key candidate genes involved in the stem growth in citrus rootstocks, such as GH3 and INV proteins (Figure 7E). Therefore, the lncRNAs possibly influenced plant growth by regulating key genes. Our findings on lncRNA and target genes suggested a potential mechanism underlying the growth regulation of citrus rootstocks. In addition, AS is a post-transcriptional regulatory process that creates multiple mRNA transcripts from the same gene and is linked to plant growth, development, and responses to environmental cues [67,68]. The present study identified genome-wide AS events in the CC, TO, and FD transcriptomes using ONT sequencing. In line with earlier reports [67], IR was the predominant AS event in the stem of the three rootstocks, and its frequency increased in the TO and FD rootstocks. This indicated that IR might regulate citrus plant growth.

## 4. Materials and Methods

### 4.1. Plant Materials and Growth Conditions

Seeds of three citrus rootstocks—’Carrizo’ citrange (CC), trifoliate orange (TO) belonging to the small-leaf (flower) type, and ‘Flying dragon’ trifoliate orange (FD)—were germinated at 30 °C. The seedlings were then planted individually in black pots containing a mixture of fertilized peat and vermiculite. Plants were grown in a greenhouse with daily temperatures between 21 °C and 36 °C and 60% to 80% relative humidity under natural photoperiod conditions. Plant height was recorded every 15 days during the experimental period (from April to September 2020). After 180 days of development, the young stem, root, and upper part of mature leaves were collected from all plants. Then, plant weight, node number, and node length were measured. Three biological replicates including 30 plants were produced for each rootstock. Upon measurement, all tissues were immediately submerged in liquid nitrogen and stored at −80 °C.

### 4.2. Measurement of Phytohormone

The contents of IAA, tZ, GA3, and ABA were determined by high-performance liquid chromatography (HPLC; E2695, Waters Corp., Milford, MA, USA) coupled with a fluorescent detector, as described by Yang et al. [69]. Briefly, the frozen samples were ground to a fine powder and extracted in 80% methanol (*v*/*v*) at 4 °C. After centrifugation, the supernatant was collected and concentrated to the aqueous phase by incubating in a 40 °C rotary evaporator (R210, BUCHI Labortechnik AG, Switzerland). The organic phase was treated with 80% methanol (*v*/*v*) and centrifuged again. After collecting, the supernatant was divided into two equal parts: one part was used for measuring IAA, GA, and ABA, and the other part for tZ. The portion for IAA, GA, and ABA isolation was adjusted to pH 2–3, then extracted twice with an equal volume of ethyl acetate, and finally evaporated to dryness with a rotary evaporator at 40 °C. The tZ was extracted twice with an equal volume of n-butanol-saturated phosphate buffer (pH 7–8) and evaporated to dryness as mentioned above. The dried samples containing IAA, tZ, and ABA were dissolved in 100% methanol (A) with 1% glacial acetic acid (B) as the mobile phase. The dried GA3 sample was dissolved in 100% acetonitrile (A) with 1% phosphoric acid (B) as the mobile phase. A compass C18 column was used to elute the phytohormones, and the detection wavelength was 275, 270, 210, and 254 nm for IAA, tZ, GA3, and ABA, respectively. The flow rate for all analyses was adjusted to 1 mL/min and the column temperature was set at 35 °C. Triplicate samples were run for each rootstock.

### 4.3. Quantification of Non-Structural Carbohydrates

Contents of non-structural carbohydrates, including sucrose, fructose, and glucose, were quantified by the HPLC system (LC-20AT, Shimadzu, Kyoto, Japan) as previously described [70]. The total starch content was determined by spectrophotometry (UV-2600, Shimadzu, Kyoto, Japan), and the measurement was performed using a starch reagent kit (G0551F) according to the manufacturer protocol (Suzhou Grace Biotechnology Co., Ltd., Suzhou, China).

### 4.4. Determination of Lignin, Cellulose, and Hemicellulose Contents

The lignin content was measured using the acetyl bromide method on a UV-2600 spectrophotometer (Shimadzu, Kyoto, Japan) at a wavelength of 280 nm [71]. The contents of cellulose and hemicellulose were determined using the assay kits (Suzhou Grace Biotechnology Co., Ltd., Suzhou, China) by sulfuric acid anthrone colorimetric method and hydrochloric acid hydrolysis method, respectively.

### 4.5. RNA Extraction, Library Construction, and Transcriptome Sequencing

Total RNA of the nine stem samples of the CC, TO, and FD rootstocks (three biological replicates for each rootstock) was isolated using TRIzol reagent (Takara, Dalian, China) according to the manufacturer instruction. The cDNA libraries were generated using the ONT protocol. In brief, the full-length mRNA reverse transcription was carried out using SuperScript IV First-Strand Synthesis System (Invitrogen, California, USA), following cDNA PCR for 14 circles with LongAmp Tag (NEB, Massachusetts, USA). The PCR products were then subjected to FFPE DNA repair and end-repair (NEB) steps and following adaptor ligation using T4 DNA ligase (NEB). The library fragments were purified with Agencourt XP system (Beckman Coulter, California, USA). Finally, the cDNA libraries were added to FLO-MIN109 flow cells and sequenced by Biomarker Technology Company (Beijing, China) on a PromethION platform.

Raw reads were first filtered by removing reads with quality score < 6 and length < 200 bp. Ribosomal RNA was also filtered by mapping to the rRNA database. Next, we searched for primers at both ends of reads to determine the full-length, non-chimeric (FLNC) transcripts. Finally, clusters of FLNC transcripts were identified after mapping to the *Poncirus trifoliata* genome sequence (http://citrus.hzau.edu.cn/, accessed on April 2022) using mimimap2, and consensus isoforms were obtained after polishing within each cluster by pinfish software.

The RNA-seq data were deposited in the Genome Sequence Archive in National Genomics Data Center, China National Center for Bioinformation/Beijing Institute of Genomics, Chinese Academy of Sciences, under the accession number CRA008644 (https://ngdc.cncb.ac.cn/gsa, accessed on November 2022).

### 4.6. Differentially Expressed Transcripts (DEGs) Identification and Functional Enrichment Annotation

Full-length reads were mapped to the reference transcriptome sequence. Reads with match quality above 5 were further used to quantify. Reads per gene/transcript per 10,000 reads mapped were used in calculating gene/transcript expression levels [72]. Differential expression analysis among the three rootstocks was implemented in the DESeq2 R package. The threshold for differentially expressed genes (DEGs) was set as FDR < 0.01 and |log2 fold change| ≥ 1. For gene function annotation, the DEGs were subjected to GO and KEGG enrichment analysis using the GOseq R packages and KOBAS software, respectively.

### 4.7. Identification of Alternative Splicing Events

The AS events, including IR, A5SS, A3SS, ES, and MEE were identified using the AStalavista tool (http://astalavista.sammeth.net/, accessed on April 2022).

### 4.8. TF Prediction and lncRNAs Analysis

The iTAK was used for plant TFs prediction [73]. Transcripts with more than 200 nt and two exons were assigned as lncRNAs candidates. We further screened novel lncRNAs by searching against the Coding Potential Calculator (CPC), Coding-Non-Coding Index (CNCI), Coding-Potential Assessment Tool (CPAT), and Protein family (Pfam) databases that can characterize non-protein coding RNA candidates from putative protein-coding RNAs among the candidate transcripts.

### 4.9. Statistical Analysis

For statistical analysis, mean ± standard error was calculated from at least three samples. All data were subjected to analysis of variance (ANOVA) using SPSS (Chicago, IL, USA). The differences between the means were conducted using Duncan’s multiple range tests (*p* < 0.05).

## 5. Conclusions

This study compared carbohydrate and hormone changes and performed a full-length transcriptome analysis in the stem of the weak TO and FD rootstocks compared with the vigorous CC rootstocks to explore the mechanism underlying dwarfing phenotype. It found that phytohormone metabolism or signaling processes of TO and FD rootstocks were disturbed by activating or inhibiting the expression of many genes such as *Aux/IAA*, *SAUR*, *GH3*, *GA2ox*, *GID1,* and *DELLA*, which were correlated with an increase in IAA, tZ, and ABA contents and a decrease in the bioactive GA content. Our results also demonstrated that the *SUS*, *INV*, *FRK*, *TPS*, and *AMS* genes related to sugar degradation, and *EG, BGL, XYL, XGOAT,* and *XTH* genes related to cellulose and hemicellulose hydrolysis were activated whereas *PAL*, *CAD*, and *COMT* genes involved in lignin biosynthesis were reduced in the TO and FD rootstocks, which were the key cause for their weak growth vigor. Moreover, many regulators, including TFs, lncRNAs, and AS events involved in the synergistic regulation of stem elongation and development in the TO and FD rootstocks were identified. The findings of this study might improve our understanding of the molecular mechanisms of regulating dwarfing traits of citrus rootstocks, thus laying a foundation for basic research and breeding application in the citrus industry.

## Data Availability

Not applicable.

## Supplementary Materials

The following supporting information can be downloaded at: https://www.mdpi.com/article/10.3390/plants12020271/s1, Figure S1: GO annotation of the DEGs common to TO and FD; Figure S2: KEGG pathway classification of the DEGs common to TO and FD; Table S1: Summary of the transcriptome data generated by Nanopore sequencing. Table S2. Character of AS events identified in this study; Table S3: Identification of transcription factors; Table S4: Analysis of long noncoding RNAs and their target genes.
