# Supplementary material for: Physiological and Full-Length Transcriptome Analyses Reveal the Dwarfing Regulation in Trifoliate Orange (Poncirus trifoliata L.)
Source: Plants (Basel). 2023 Jan 6;12(2):271. doi: 10.3390/plants12020271 (PMC9860739; doi:10.3390/plants12020271)
Supplement: Supplementary file 1 [file plants-12-00271-s001.zip › Table S1.pdf]

Table S1. Summary of the transcriptome data generated by Nanopore sequencing.

	Reads number	N50	Mean length	Max length	Clean reads number	Full-length reads number	Full-length percentage	Mapped reads	Mapped rates
CC1	3,435,847	1,262	1,112	13,786	3,424,731	2901338	84.72%	2,859,934	98.57%
CC2	3,711,466	1,263	1,113	13,286	3,699,975	3176184	85.84%	3,131,759	98.60%
CC3	3,900,771	1,239	1,095	13,188	3,885,152	3366912	86.66%	3,318,532	98.56%
TO1	4,720,308	1,219	1,080	15,453	4,708,160	3963437	84.18%	3,925,208	99.04%
TO2	4,490,517	1,224	1,083	13,138	4,479,497	3778870	84.36%	3,742,813	99.05%
TO3	4,090,332	1,236	1,092	18,286	4,080,212	3459687	84.79%	3,427,580	99.07%
FD1	3,753,973	1,188	1,058	26,714	3,743,130	3261142	87.12%	3,231,633	99.10%
FD2	4,308,872	1,177	1,050	13,410	4,294,422	3742087	87.14%	3,706,669	99.05%
FD3	4,364,220	1,210	1,072	12,023	4,350,039	3778237	86.86%	3,744,204	99.10%
