# Supplementary figures and images for: Physiological and Full-Length Transcriptome Analyses Reveal the Dwarfing Regulation in Trifoliate Orange (Poncirus trifoliata L.)
Source: Plants (Basel). 2023 Jan 6;12(2):271. doi: 10.3390/plants12020271 (PMC9860739; doi:10.3390/plants12020271)

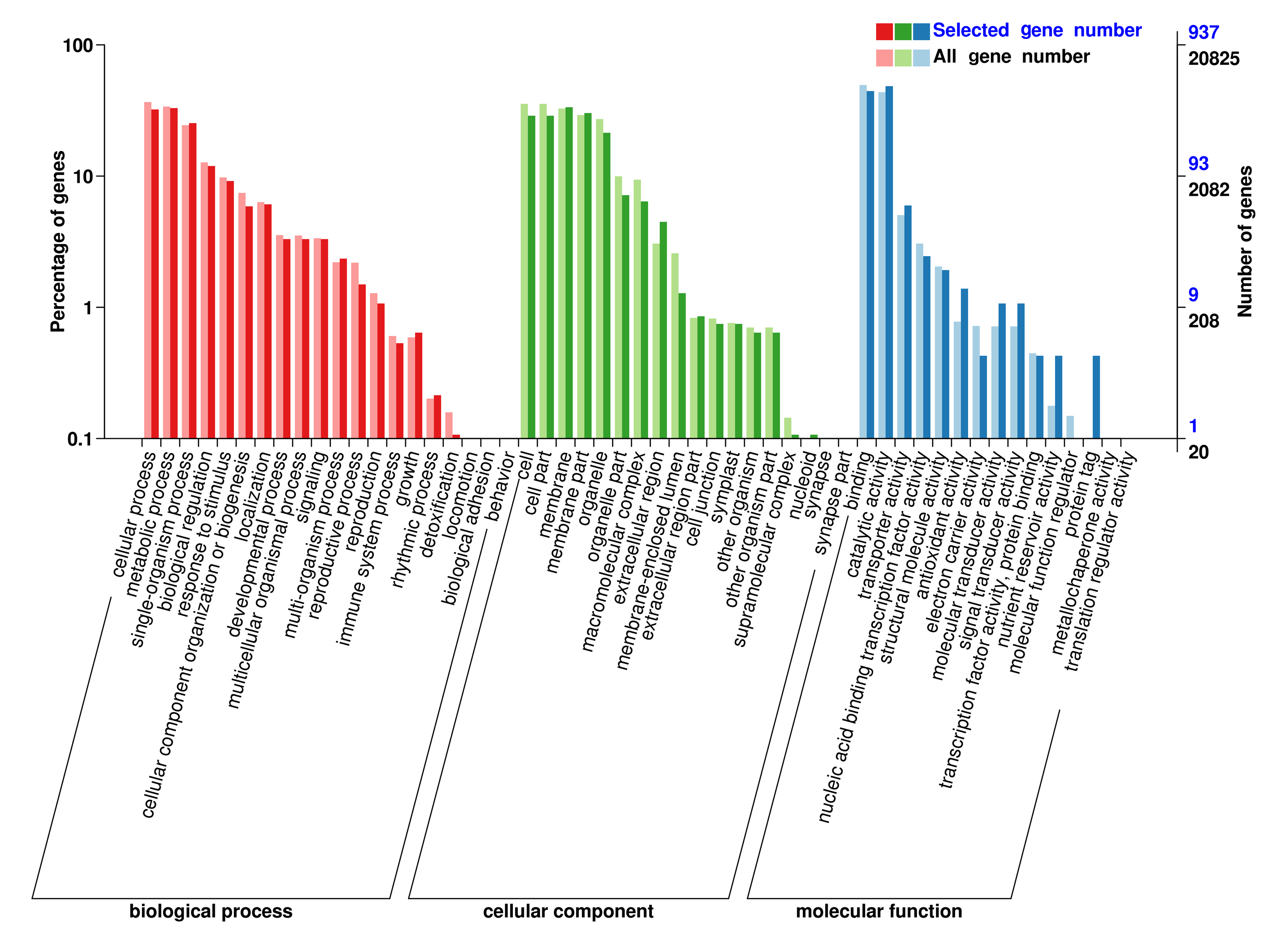

Supplement: Supplementary file 1 [file plants-12-00271-s001.zip › Fig.S1.tif]

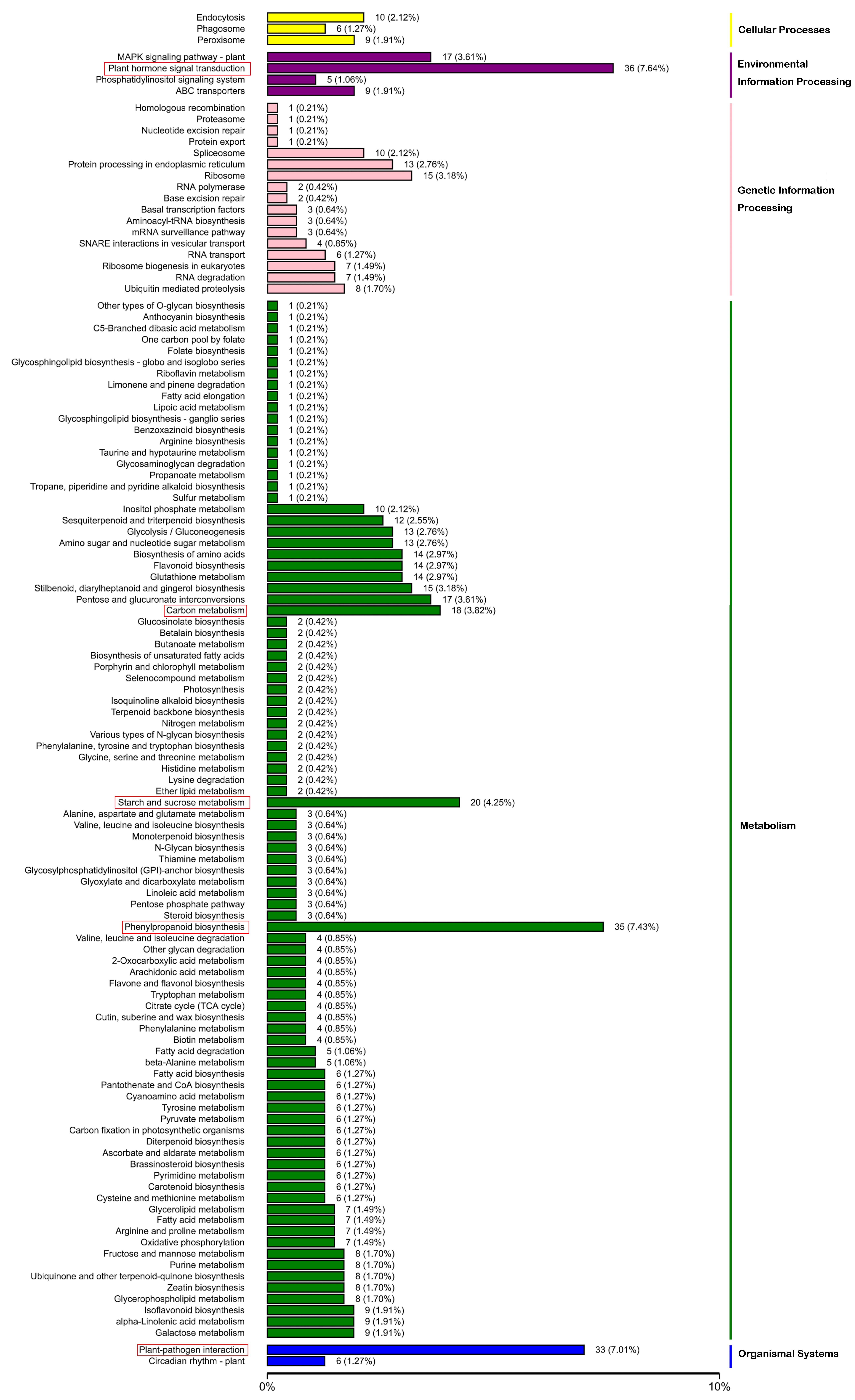

Supplement: Supplementary file 1 [file plants-12-00271-s001.zip › Fig.S2.tif]
